# Protocol for chromatin immunoprecipitation of histone modifications in frozen adipose tissue

**DOI:** 10.1016/j.xpro.2024.103163

**Published:** 2024-06-27

**Authors:** Akin Cayir, Tone M. Tannæs, Sadia Saeed, Matthias Blüher, Yvonne Böttcher

**Affiliations:** 1 EpiGen, Medical Division, Akershus University Hospital, 1478 Lørenskog, Norway; 2 Department of Clinical Molecular Biology, EpiGen, Institute of Clinical Medicine, University of Oslo, 1171 Oslo, Norway; 3 Helmholtz Institute for Metabolic, Obesity and Vascular Research (HI-MAG) of the Helmholtz Zentrum München at the University of Leipzig and University Hospital, Leipzig, Germany

**Keywords:** Chromatin immunoprecipitation (ChIP), Health Sciences

## Abstract

Chromatin immunoprecipitation (ChIP) combined with sequencing has revolutionized our understanding of gene regulation; however, its application to frozen adipose tissue presents unique challenges due to the high levels of lipid content. Here, we present a protocol for ChIP of histone modifications in human frozen adipose tissue. We describe steps for tissue preparation, chromatin isolation, sonication, pre-clearing of chromatin, and immunoprecipitation. We then detail procedures for elution, crosslink reversal, chromatin purification, quality control, and library synthesis.

## Highlights

• Utilizes a small input of chromatin from frozen human adipose tissues
• Achieves successful immunoprecipitation with efficient washing steps
• Ensures high-quality ChIP material after elution and de-crosslinking steps
• Optimizes all conditions to produce high-quality sequencing data

## Abstract (editor highlights)

Publisher’s note: Undertaking any experimental protocol requires adherence to local institutional guidelines for laboratory safety and ethics.

## Graphical abstract

**Figure:**
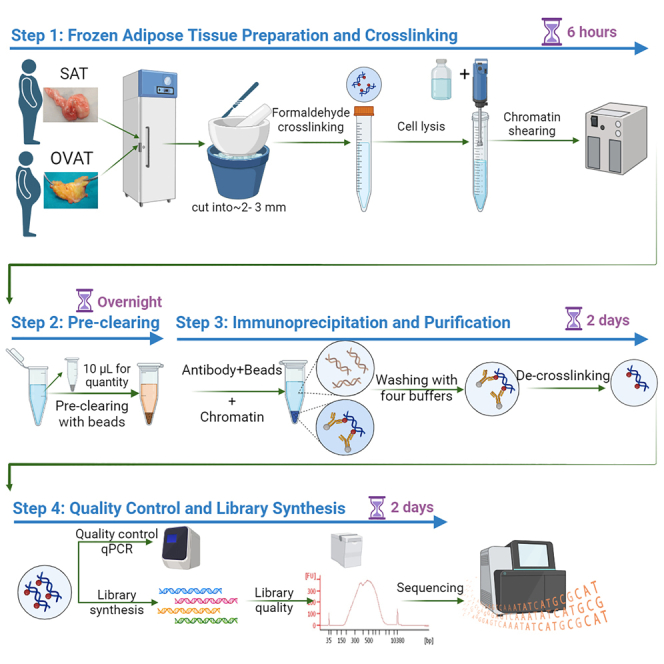

## Before you begin

A well-refined chromatin immunoprecipitation (ChIP) protocol is essential for acquiring sequencing data of high quality. This protocol should be adaptable to diverse adipose tissue types, encompassing various depots, and capable of yielding ChIP material of exceptional quality for sequencing across different histone modifications. In case of adipose tissue, and especially in frozen adipose tissue, the ChIP protocol to analyze the histone modifications presents significant challenges. Importantly, adipose tissue poses unique difficulties due to its heterogeneous cellular composition for both subcutaneous adipose tissue (SAT) and omental visceral adipose tissue (OVAT) comprising adipocytes, pre-adipocytes, mesenchymal cells, immune cells, endothelial cells and many more.^1^ Additionally, the high lipid content creates challenges to optimal chromatin immunoprecipitation for both fresh and frozen adipose tissue that historically required high input of chromatin.^2^ Here, we optimize multiple experimental steps that result in small chromatin input along with short application time and ensuring high quality sequencing data (unpublished data).

### Institutional permissions

This study is approved by the Regional Ethics Committee in the Health Region South-East Norway (REK 99047).

## Key resources table

**Table:** 

REAGENT or RESOURCE	SOURCE	IDENTIFIER
**Antibodies**		

Histone H3K36me3 antibody (50 μg)	Active Motif	Cat#61022

**Biological samples**		

Human frozen SAT and OVAT samples	N/A	N/A

**Chemicals, peptides, and recombinant proteins**		

Pierce 16% formaldehyde (w/v), methanol-free (FA)	Thermo Fisher Scientific	Cat#28906
cOmplete, EDTA-free protease inhibitor cocktail (PIC)	Merck-Roche	Cat#11873580001
Sodium butyrate (NaBu)	Sigma-Aldrich	Cat#B5887-1G
Proteinase K	QIAGEN	Cat#74004
Sodium dodecyl sulfate (SDS)	Bio-Rad	Cat#161-0418
RNase A	Sigma-Aldrich	Cat#R6148
Sodium acetate buffer solution (NaAc)	Sigma-Aldrich	Cat#S7899
Nonidet P-40 substitute (NP-40)	Roche	Cat#11332473001
PIPES, pH = 8.0	Alfa Aesar	Cat# J60618
TE (Tris-EDTA) buffer (pH = 8.0)	VWR, Life Science	Cat# E112
NaCl	Sigma-Aldrich	Cat#S5150
LiCl (8 M)	Sigma-Aldrich	Cat#L7026
Phenylmethanesulfonyl fluoride (PMSF)	Merck-Roche	Cat#P7626

**Critical commercial assays**		

High-sensitivity DNA kit – 10 chips	Agilent Technologies	Cat#5067- 4626
DNA LoBind tubes	Eppendorf (1.5 mL)	Cat#30108051
MinElute PCR purification kit (250)	QIAGEN	Cat#28006
Qubit dsDNA HS kit (100)	Thermo Fisher Scientific	Cat#Q32851
SMARTer ThruPLEX DNA-Seq kit 48 rxns	Takara/AH Diagnostics	Cat#R400675
SMARTer DNA unique dual index kit-set B,	Takara/AH Diagnostics	Cat#R400666
AMPure XP beads, 60 mL	Beckman Coulter	Cat#A63881
PowerUp SYBR green master mix 2 × 5 mL	Thermo Fisher Scientific	Cat#A25742
Dynabeads protein G for immunoprecipitation	Thermo Fisher Scientific	Cat#10003D-1G

**Oligonucleotides**		

*Actin*, F:5′GCTGTTCCAGGCTCTGTTCC 3′	Thermo Fisher Scientific	N/A
*Actin*, R:5′GCTCACACGCCACAACATG 3′	Thermo Fisher Scientific	N/A
*ADIPOQ,* F*:* 5′AGACATCGGTGAAACCGGAG 3′	Thermo Fisher Scientific	N/A
*ADIPOQ,* R*:* 5′ACACTGAATGCTGAGCGGTA 3′	Thermo Fisher Scientific	N/A
Human negative control primer set 1	Active Motif	Cat#71001

**Other**		

DynaMag-2 magnet	Invitrogen	Cat#12321D
Homogenizer: Omni Tissue Homogenizer (TH) with hard tissue Omni Tip plastic homogenizing probes (7 mm × 110 mm) – 1,000 pack (omni-inc.com) (OMNI)	Omni Tissue Homogenizer	N/A
Sonicator: Bioruptor plus	Diagenode	UCD-300
Rotator (rotating wheel) with adjustable speed, placed in a cold room	Stuart	SRT6D
Table-top centrifuge with a swing-out rotor for 1.5-mL tubes.	VWR	Micro Star 21R
Centrifuge with a swing-out rotor suited for 15-mL conical tubes	Thermo Fisher Scientific	Microfuge X3R
Vortex	IKA	N/A
Thermomixer	Eppendorf 1.5 mL	N/A
Agarose gel electrophoresis apparatus.	Bio-Rad	N/A
Qubit spectrophotometer	Thermo Fisher Scientific	Qubit Flex Fluorometers
Thermal cycler with real-time capacity	Thermo Fisher Scientific	QuantStudio 7Flex3
Laminar flow hood (LAF) bench	Herasafe	Cat#2030

## Materials and equipment

### Buffers

***Alternatives:*** All the reagents used in this protocol can be replaced with the same function from different suppliers.

### Chemical solution

• 0.5 M Tris-Hydrochloride (HCL) pH = 8.1 and pH = 8 (3.03 g Tris Base, to 50 mL with ddH2O).
• Tris-HCl (pH 7.5) (0.5 M) pH: 7.5 (3.03 g Tris-Base, to 50 mL with ddH2O).
• 0.1 M EDTA pH = 8 (1.86 g EDTA, to 50 mL with ddH2O).
• 0.1 M EGTA (1.90 g EGTA, to 50 mL with ddH2O).
• 2.5 M Glycine (9.38 g Glycine, to 50 mL with ddH2O).
• Sodium deoxycholate (100%) (5 g Sodium deoxycholate, to 5 mL with ddH2O).
• 500 ng/μL RNase A (2.5 μL RNase A from 20 mg/mL, to 100 μL with ddH2O).
• 2 μg/ μL Proteinase K (1 μL Proteinase K, to 10 μL ddH2O).
• 50× PIC (1 tablet + 1 mL ddH2O).
• 50 mM PMSF (87.10 mg PMSF, to 10 mL with ddH2O).
• 1 M NaBu (1.10 g NaBu, to 10 mL with ddH2O).
• 1 M KCl (745.51 mg KCl, to 10 mL with ddH2O).
• 1% FA (312.5 μL FA, to 5 mL with PBS).
• 1 M LiCl (1 mL LiCl, to 8 mL with ddH2O).

***Note:*** If necessary, use either 1 M Hydrochloric acid or 1 M NaOH solutions for pH adjustment.

### RIPA zero-SDS buffer

Store at 4°C for up to 6 months.

**Table:** 

Reagents	Final concentration	Amount
Tris-HCl (pH 8.0) (0.5 M)	10 mM	10 mL
NaCl (5.0 M)	140 mM	14 mL
EDTA (0.1 M)	1 mM	5 mL
EGTA (0.1 M)	0.5 mM	2.5 mL
Sodium deoxycholate (100%)	0.1% (wt/vol)	0.5 mL
Triton X-100 (100%)	1% (vol/vol)	5 mL
SDS (10%)∗	0.1%	5 mL
PIC (50×)∗	1×	10 mL
PMSF (50 mM)∗	1 mM	10 mL
NaBu (1 M)∗	20 mM	10 mL
Nuclease-free water	N/A	428 mL
**Total**	**N/A**	**500 mL**

∗ Add just before use

### Cell lysis buffer

Prepare immediately before the experiment and store it on ice.

**Table:** 

Reagents	Final concentration	Amount
PIPES (1 M)	5 mM	25 μL
KCl (1 M)	85 mM	425 μL
NP-40 (10%)	1%	500 μL
PIC (50×)	1×	100 μL
PMSF (50 mM)	1 mM	100 μL
NaBu (1 M)	20 mM	100 μL
Nuclease-free water	N/A	3750 μL
**Total**	**N/A**	**5000 μL**

### Nuclei lysis with SDS

Prepare immediately before the experiment and store it on ice.

**Table:** 

Reagents	Final concentration	Amount
Tris pH 8.1 (0.5 M)	50 mM	30 μL
EDTA (0.1 M)	10 mM	30 μL
SDS (10%)	1%	30 μL
PIC (50×)	1×	6 μL
PMSF (50 mM)	1 mM	6 μL
NaBu (1M)	20 mM	6 μL
Nuclease-free water	N/A	192 μL
**Total**	**N/A**	**300 μL**

### Nuclei lysis without SDS

Prepare immediately before the experiment and store it on ice.

**Table:** 

Reagents	Final concentration	Amount
Tris pH 8.1 (0.5 M)	50 mM	30 μL
EDTA (0.1 M)	10 mM	30 μL
PIC (50×)	1×	6 μL
PMSF (50 mM)	1 mM	6 μL
NaBu (1M)	20 mM	6 μL
Nuclease-free water	N/A	222 μL
**Total**	**N/A**	**300 μL**

### Elution buffer

Prepare freshly.

**Table:** 

Reagents	Final concentration	Amount
NaCl (5.0 M)	50 mM	10 μL
Tris-HCl (pH 7.5) (0.5 M)	20 mM	40 μL
EDTA (pH 8.0) (0.1 M)	5 mM	50 μL
NaBu (1.0 M)	20 mM	20 μL
Nuclease-free water	N/A	780 μL
SDS (10%)	1%	100 μL, Add just before use
**Total**	**N/A**	**1000 μL**

### Wash buffer 1

Store at 4°C for up to 12 months.

**Table:** 

Reagents	Final concentration	Amount
RIPA zero-SDS buffer	N/A	930 μL
SDS (10%)∗	0.1%	10 μL
PIC (50×)∗	1×	20 μL
PMSF (50 mM)∗	1 mM	20 μL
NaBu (1M)∗	20 mM	20 μL
**Total**	**N/A**	**1000 μL**

∗ Add just before use

### Wash buffer 2

Store at 4°C for up to 12 months.

**Table:** 

Reagents	Final concentration	Amount
Tris-HCl (pH 8.1) (0.5 M)	20 mM	40 μL
NaCl (5.0 M)	500 mM	100 μL
EDTA (0.1 M)	2 mM	20 μL
Triton X-100 (100%)	1% (vol/vol)	10 μL
SDS (10%)∗	0.1%	10 μL
PIC (50×)∗	1×	20 μL
PMSF (50 mM)∗	1 mM	20 μL
NaBu (1M)∗	20 mM	20 μL
Nuclease-free water	N/A	760 μL
**Total**	**N/A**	**1000 μL**

∗ Add just before use

### Wash buffer 3

Store at 4°C for up to 12 months.

**Table:** 

Reagents	Final concentration	Amount
Tris-HCl (pH 8.1) (0.5 M)	10 mM	20 μL
Deoxycholate (100%)	1%	10 μL
EDTA (0.1 M)	1 mM	10 μL
NP-40 (100%)	1% (vol/vol)	10 μL
LiCl (1 M)	0.25 M	25 μL
SDS (10%)∗	0.1%	10 μL
PIC (50×)∗	1×	20 μL
PMSF (50 mM)∗	1 mM	20 μL
NaBu (1M)∗	20 mM	20 μL
Nuclease-free water	N/A	855 μL
**Total**	**N/A**	**1000 μL**

∗ Add just before use

### TE buffer

Prepare freshly.

**Table:** 

Reagents	Final concentration	Amount
Tris-HCl (pH 8.1) (0.5 M)	10 mM	20 μL
EDTA (0.1 M)	1 mM	10 μL
Nuclease-free water	N/A	970 μL
**Total**	**N/A**	**1000 μL**

### Equipment

• HERASAFE LAF bench.

***Alternatives:*** Any LAF bench is suitable to work during FA

• Invitrogen DynaMag-2 Magnet, Invitrogen.

***Alternatives:*** Any magnetic rack is suitable for magnetic separation

• Omni Tissue Homogenizer.

***Alternatives:*** Any homogenizer to disrupt the soft tissues such as Benchmark Scientific D1000 Handheld Homogenizer

• Bioruptor Plus Diagenode Sonicator.

***Alternatives:*** E220 Focused-ultrasonicator, Covaris

• Stuart Rotator (rotating wheel).

***Alternatives:*** Any rotator with adjustable time and rotating speed

• VWR centrifuge with a swing-out rotor for 1.5-mL tubes.

***Alternatives:*** Any centrifuge is suitable for 1.5 mL tubes with cooling system

• Thermo Fisher Scientific Centrifuge with a swing-out rotor suited for 15-mL conical tubes.

***Alternatives:*** Any centrifuge is suitable for 1.5 mL tubes with cooling system

• IKA Vortex.

***Alternatives:*** Any vortex is suitable

• Eppendorf 1.5 mL Thermomixer.

***Alternatives:*** Any mixer with controlling temperature and time

• BIO-RAD Agarose gel electrophoresis apparatus.

***Alternatives:*** Any gel system

• Thermo Fisher Scientific Qubit spectrophotometer.

***Alternatives:*** NanoDrop 2000 Spectrophotometer, Thermo Fisher Scientific

• QuantStudio 7Flex3 thermal cycler with real-time capacity.

***Alternatives:*** Any qPCR machine

## Step-by-step method details

### Preparation of adipose tissues for the fixation

**Timing: 120 min**

This protocol is designed for a single ChIP assay targeting H3K36me3. It has been optimized for different human frozen adipose tissue depots, specifically SAT and OVAT, which were snap frozen and stored at −80°C post-surgery. In this step, we prepare frozen adipose tissues for the fixation.

**CRITICAL:** Cut the tissue in a mortar using a pre-chilled scalpel on dry ice.

#### Processes

1. Take the samples from −80°C into dry ice.
2. Put tissue samples in the pre-cooled mortar with dry ice.
  a. Place sarstedt screw cap tubes (15 mL) are in dry ice.
  b. By using a cold scalpel, cut tissue samples into smaller pieces (∼2–3 mm).
3. Add 3 mL of 1% FA (fresh) in PBS (Ca/Mg free) to each tube containing approximately 100 mg adipose tissue.
  a. Put on slight rotation at 30 rpm at room temperature for 10 min, avoiding from direct light.

**CRITICAL:** Weight adipose tissues just before experiment.

4. Cross-linking is stopped by adding 158 μL of Glycine (2.5 M) in PBS.

***Note:*** The final concentration of Glycine is 0.125 M.

5. Continue rotating at room temperature for 10 min at 30 rpm at room temperature.
6. Centrifuge the tubes at 470 g at 4°C for 10 min at COLD ROOM (+4°C).
7. Following centrifugation, remove gently the liquid phase by a glass pipette.

***Note:*** There may be two layers of tissue observed either settling at the bottom of the tube or forming a top layer. Some samples may exhibit tissue cuts appearing to float within the liquid. The primary objective here is to eliminate the liquid phase containing FA and Glycine while ensuring that small tissue fragments remain untouched. It is essential to maintain the samples on ice throughout this process.

8. Wash the samples with the resulting tissue pellet and the tissue from the top layer three times with 5 mL of cold PBS with PIC (20 μL per 1 mL).
9. Centrifuge tubes at 470 g at 4°C for 10 min.
  a. The liquid phase is removed by a glass pipette.

***Note:*** Work in the hood.

### Chromatin isolation and sonication

**Timing: 240 min**

Here, we isolate chromatin and apply sonication process for the chromatin fragmentation.

***Note:*** Add PIC, NaBu, and PMSF just before use.

***Note:*** Maintain all buffers and samples on ice during this stage.

***Note:*** Prepare nucleus lysis buffer, with and without SDS, which we used to adjust the final SDS concentration to 0.5%.

10. Add 5 mL of cold cell lysis buffer with PIC, NaBu, and PMSF to each tube from step 9 (on ice).
11. Homogenize the tissue by an OMNI homogenizer with Hard Tissue Omni Tip (blue)

***Note:*** 3 times 10 sec with a 30-sec break on ice in between.

12. Incubate tubes on ice for 30 min while vortexing (10 s) every 3 min.
13. After incubation, centrifuge at 2870 g for 10 min at 4°C to precipitate the resulting cell pellet.
14. Remove the supernatant by inverting the tube.

***Note:*** The tube can be left upside down on paper to avoid lipids running down onto the pellet.

15. Add 1 mL of cell lysis buffer with PIC, NaBu, and PMSF to each tube. Mix by vortexing and centrifuge at 2,870 g for 10 min at 4°C

***Note:*** This process enables to precipitate the core fraction, which can help to remove excess fat.

16. Re-suspend the nuclear pellet in 150 μL cold nucleus lysis buffer (plus 1.0% SDS) with PIC, NaBu, and PMSF and transfer to a ‘sonication’ tube (1.5 mL).
17. Vortex and incubate the tubes for 2 h on ice.
18. After 2 h, add 150 μL cold nucleus lysis buffer without SDS.

***Note:*** This is required for shearing chromatin at the optimal size range. Thus, the final SDS concentration is 0.5%. The total volume of the chromatin solution is 300 μL.

19. After incubation on ice, proceed with chromatin shearing using Bioruptor UCD-300 (Diagenode).

**Figure 1:**
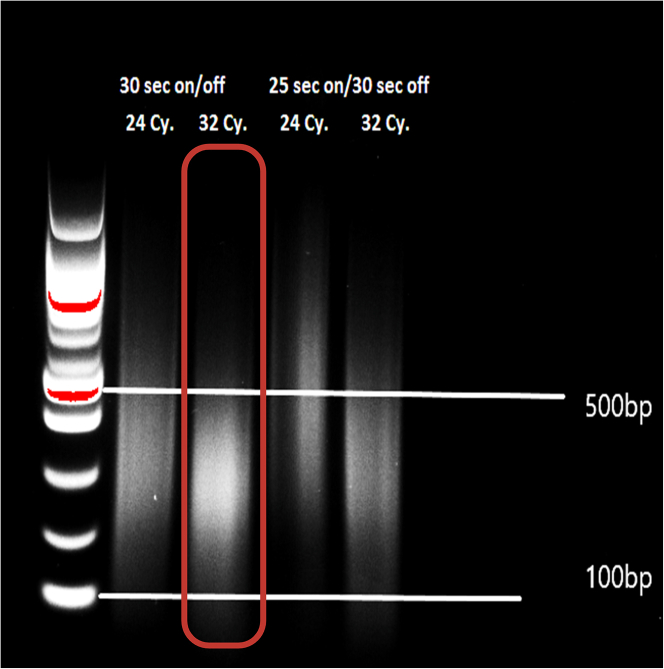
An example of different sonication conditions (Cy: cycle)

***Note:*** Use the following settings: High power, 30 s ON/ 30 s OFF, 8 cycles, repeat 4 times; 32 cycles in total. After each cycle, briefly, vortex, spin and incubate for 2 min on ice (Figure 1).

20. After shearing, centrifuge tubes at 10,000 g at 4°C for 15 min.

***Note:*** Following centrifugation, the tube exhibits three distinct layers. The bottom layer comprises tissue debris, while the top layer appears blurry and is thinner. Positioned between these layers is a transparent layer containing chromatin.

21. Collect transparent layer without disturbing the bottom and top layers and transfer it to DNA low-binding tubes (1.5 mL).
22. Take 10 μL of chromatin for quality and quantity control.

#### Pre-clearing of chromatin

**Timing: Overnight**

Here, we pre-clear the chromatin by incubating it with the beads.

23. A pre-clearing step can be performed here before storing the chromatin at −80°C.

***Note:*** This is optional and pre-clearing of the chromatin can also be performed directly before immunoprecipitation.

24. Wash 10 μL of protein-G dynabeads with 100 μL of RIPA Buffer containing 0.1% SDS three times.
25. Allow a 2 min incubation period on ice, briefly spin, place the tube on a magnetic plate, and then remove the buffer by pipetting.
26. Following the last wash, without resuspending in any buffer again,
  a. Add isolated chromatin (100 μL) immediately to protein G dynabeads beads (10 μL) within 1.5 mL low-binding DNA tubes
  b. Rotate overnight at COLD ROOM (+4°C).
27. Perform a quick spin to collect the liquid that is trapped in the cap.
  a. Place the tube containing chromatin and beads mix in a magnetic rack to hold 1.5 mL tubes.
  b. Wait for 2 min.
  c. Take the buffer containing pre-cleared chromatin.
  d. Put it in a new 1.5 mL low-binding DNA tube

***Note:*** Place magnetic plate in ice during pre-clearing processes

28. Store pre-cleared chromatin at −80°C without snap freeze.

### Quality and quantity control of chromatin

**Timing: 105 min**

Here, we prepare the chromatin to determine the quality and quantity.

29. Take 10 μL of the pre-cleared chromatin from step 28.
  a. Add 1 μL RNase A (from 500 ng/μL stock).
  b. Incubate at 37°C for 20 min at 500 rpm using Thermomixer.
30. Prepare elution buffer and add 190 μL of fresh elution buffer (add 1% SDS before use).
31. Add 5 μL proteinase K from a 10-fold dilution of the 20 mg/mL stock and incubate at 37°C for 20 min.
32. Incubate at 68°C at 500 rpm for 1 h.

### Purification and analysis of chromatin fragment size

**Timing: 90 min**

Here, we isolate chromatin and determine the fragment size.

33. Purify the chromatin with MinElute PCR purification kit.
34. Add 5 volumes (1025 μL) of binding buffer (PB) to the sample.
  a. Add 40 μL of 3 M NaAc (pH: 5.2).
35. Add 700 μL of the sample in MinElute column.
  a. Centrifuge 1 min at 13,000 rpm.
  b. Remove flow through.
36. Load the rest of the samples in the same column until loading all the samples.
  a. Centrifuge 1 min at 13,000 rpm.
  b. Remove flow through.
37. Wash with 700 μL of PE buffer.
  a. Centrifuge 1 min at 13,000 rpm.
  b. Remove flow through.
38. Put the samples in a new collection tube and centrifuge for 1 min at 13,000 rpm.
39. Put MinElute column in 1.5 mL low-DNA binding tube.
  a. Add 10 μL TE (Tris-EDTA) Buffer (pH = 8.0).
  b. Incubate for 1 min at 37°C.
  c. Centrifuge 1 min at 13,000 rpm.
40. Maintain the samples at 55°C for 5–10 min to allow ethanol evaporation beforehand.

***Note:*** To remove any residual alcohol from the reaction.

41. Use Qubit dsDNA HS kit and determine the dsDNA concentration by Qubit fluorometer.
  a. Prepare the standards used in the calibration.
  b. Prepare working solution.
  c. Add 1 μL of DNA to working solution.
  d. Measure the concentration.
42. Add 1 μL gel-pilot loading (5×, QIAGEN) dye + 5 μL of chromatin (200–250 ng in total).
  a. Run at 90–100 V (45–60 min).
  b. Analyze DNA in a 1.5% agarose gel with suitable size markers (1000 bp) to determine fragment size.

***Alternatives:*** 2100 Bioanalyzer system (Agilent, USA) is an alternative approach for the fragment size and distribution. Briefly, prepare the gel-dye mix by high sensitivity DNA dye with high sensitivity DNA gel matrix. Load 9 μL of the gel-dye mix in wells in high sensitivity DNA chip as indicated in the protocol. Load 5 μL of green- capped high sensitivity DNA marker into the wells marked with the ladder symbol. Load the 1 μL of ladder and the samples, put in vortex mixer and start the chip run.

***Alternatives:*** Nanodrop offers an alternative approach, consistently providing concentration measurements that are higher than those obtained with the Qubit due to a lack of sensitivity. Qubit provides high sensitivity, whilst Nanodrop provides purity measurements.

### Immunoprecipitation step

#### Coupling of dynabeads with histone modification-specific antibody

**Timing: 135 min**

We incubate dynabeads with histone modification-specific antibody for coupling.

43. Vortex the bottle of dynabeads stock for 1 min before taking out the beads.
44. Take 10 μL of beads per ChIP in a 1.5 mL tube (low DNA binding).

***Note:*** Calculate the amount of beads you need based on the number of ChIPs.

45. Add 2.5× volumes (25 μL) of RIPA buffer (0.1% SDS) (containing PIC, NaBu, and PMSF) with gentle pipetting.
46. Incubate the beads for 1 min on ice.
  a. Place them on the magnet for 1 min.
  b. Remove the buffer by gentle pipetting.

***Note:*** To avoid beads trapped in the lid, snap-spin the tube in a mini-centrifuge before putting the tube on the magnet.

47. Repeat two times steps 45 and 46.
48. Resuspend the beads in 10× volume of RIPA buffer (100 μL) containing 0.1% SDS.
49. Add 1 μL of 2.5 μg H3K36me3 antibody to each 1.5 mL DNA low-binding tube.

***Note:*** Do gentle pipetting and do not vortexing. If requires, snap-spin the tube in a mini-centrifuge.

***Note:*** Test antibody concentrations for H3K4me3, H3K4me1, H3K27me3, and H3K27ac modifications.

50. Incubate the tubes (beads + antibody) at 40 rpm on a rotator in the COLD ROOM (+4°C) for 2 h.

***Note:*** We test that the incubation duration differ for various antibodies targeting H3K4me3, H3K4me1, H3K27me3, and H3K27ac. For those modifications, we investigate the effects of 0.5, 1, and 2 h incubation periods and subsequently compare the immunoprecipitation percentages obtained from each duration.

### Immunoprecipitation

**Timing: 135 min**

Here, we immunoprecipitate the chromatin.

51. After 2 h of incubation,
  a. Put the tube on the magnet for 1 min.
  b. Remove the buffer containing the unconjugated antibody.
52. Adding pre-cleared chromatin (∼500 ng DNA in 100 μL) and in parallel;
  a. Put 5% of chromatin as INPUT in another DNA low-binding tube.
  b. Place the input samples on ice until being processed in step 64.

***Note:*** The initial concentration of chromatin primarily determines the incubation time. Using ∼500 ng of DNA in total, necessitates a 2-h incubation with antibody-bead complex.

***Note:*** The final chromatin volume must be 100 μL. Using a larger volume might lead to increased non-specific binding.

***Note:*** If the DNA concentration is elevated, adjust both the volume and concentration using nucleus lysis buffer (with 0.5% SDS).

***Note:*** Conversely, if the DNA concentration is lower, ensure that up to 300 ng of DNA in total per ChIP can be utilized.

***Note:*** As per the protocol recommendation, when working with 500 ng of DNA, employ 25 μL to achieve a 5% proportion for input.

53. Incubate the tubes horizontally (pre-cleared chromatin + beads-antibody complex) on a rotator at 40 rpm in the cold room (+4°C) for 2 h.

### Washing of the immunoprecipitated material

**Timing: 90 min**

We wash the immunoprecipitated material to remove unspecific chromatin or debris.

54. After incubation, snap-spin the tubes in a micro-centrifuge to bring down any solution trapped in the lid.
55. Place the tubes on a magnet rack on ice for 1 min.
56. Remove the supernatant while tubes are on the magnet.
  a. Throw away the supernatant.

***Note:*** Be careful to avoid the dynabeads.

57. Add 100 μL wash buffer (WB) 1 (RIPA Buffer, add 0.1% SDS just before use).
  a. Incubate the tubes for 4 min at 40 rpm on a rotator in the cold room (+4°C).
  b. Repeat one more time.
58. Add 100 μL of WB 2 (add 0.1% SDS just before use).
  a. Incubate the tubes for 4 min at 40 rpm on a rotator in the cold room.
  b. Repeat one more time.
59. Add 100 μL of WB 3 (add 0.1% SDS just before use).
  a. Incubate the tubes for 4 min at 40 rpm on a rotator in the cold room.
  b. Repeat one more time.
60. Add 100 μL of TE buffer and incubate tubes for 4 min at 40 rpm on a rotator in the cold room.
61. Before removing the TE buffer, transfer the mixture to a new tube and place it on the magnet for 1 min remove the TE buffer.
62. Add 100 μL of TE buffer and incubate tubes for 4 min at 40 rpm on a rotator in the cold room and remove the TE (10 mM Tris-HCl pH 8.1 and 1 mM EDTA).

***Note:*** Prepare all buffers freshly and keep on ice.

### Elution and crosslink reversal

**Timing: Overnight**

We elute and de-crosslink the CHIP material from the beads.

63. Prepare elution buffer (fresh-room temperature) and add 141.25 μL of an elution buffer containing 1% SDS and 5 μL of *RNase* (500 ng/μL) to ChIP.
64. Add 282.5 μL of an elution buffer containing 1% SDS and add 10 μL of *RNase* (500 ng/μL) in INPUT (5% of chromatin).
65. Incubate ChIP and INPUT samples at 37°C, 1300 rpm rotating for 20 min using Thermomixer.
66. Add 3.75 μL and 7.5 μL of proteinase K (2 mg/mL) to ChIP and INPUT, respectively and incubate at 37°C, 1300 rpm for 20 min.
67. Incubate ChIP and INPUT at 68°C overnight at 1300 rpm on a Thermomixer for reverse cross linking.
68. After overnight incubation, snap-spin samples-beads mix, and place on the magnet for 1 min, and transfer the buffer into 2 mL low binding DNA tube.
69. Add 150 μL of elution buffer (fresh-room temperature) containing 1% SDS to the beads again and incubate at 68°C for 10 min at 1300 rpm on a Thermomixer.

***Note:*** No process for INPUT sample.

***Note:*** This is an additional step to collect all the chromatin from the beads.

70. After 10 min, snap-spin the tubes.
  a. Place the tubes on the magnet for 1 min.
  b. Add the buffer into the tubes from step 68 (into 2 mL tube) (300 μL in total).

***Note:*** We usually incubate approximately 18 h for de-crosslinking step. Less incubation results in decreased concentration of ChIP material.

### Purification of the immunoprecipitated chromatin

**Timing: 30 min**

We purify the immunoprecipitated chromatin for library synthesis.

71. Purify the DNA with MinElute PCR purification kit.
72. Add 5 volumes of PB buffer to ChIP and INPUT samples.
73. Add 40 μL of 3M NaAc (pH: 5.2).
74. Add 700 μL of the ChIP and INPUT samples in MinElute column and centrifuge 1 min at 13,000 rpm and remove flow through.
75. Load the rest of the samples in the same column until loading all the samples.
  a. Centrifuge 1 min at 13,000 rpm and remove flow through.
76. Wash with 700 μL of PE buffer.
  a. Centrifuge 1 min at 13,000 rpm.
  b. Remove flow through.
77. Put the samples in a new collection tube and centrifuge for 1 min at 13,000 rpm.
78. Put MinElute column in a 1.5 mL low-DNA binding tube.
  a. Add 10 μL TE buffer.
  b. Wait for 1 min at 37°C.
  c. Centrifuge for 1 min at 13,000 rpm.
79. Determine the concentration by Qubit fluorometer.

***Note:*** The INPUT sample concentration should be at approximately 6 ng/μL, while the concentration of the ChIP sample should range between 0.3-2 ng/μL.

***Note:*** An elevated concentration in ChIP material could suggest non-specific binding.

***Note:*** We recommend using TE buffer (pH = 8.0) for elution, as it is necessary for the subsequent library preparation.

### Quality control for a successful ChIP Assay-qPCR

**Timing: 180 min**

We run RT-qPCR to control the enrichment in positive and negative control genomic regions.

**Figure 2:**
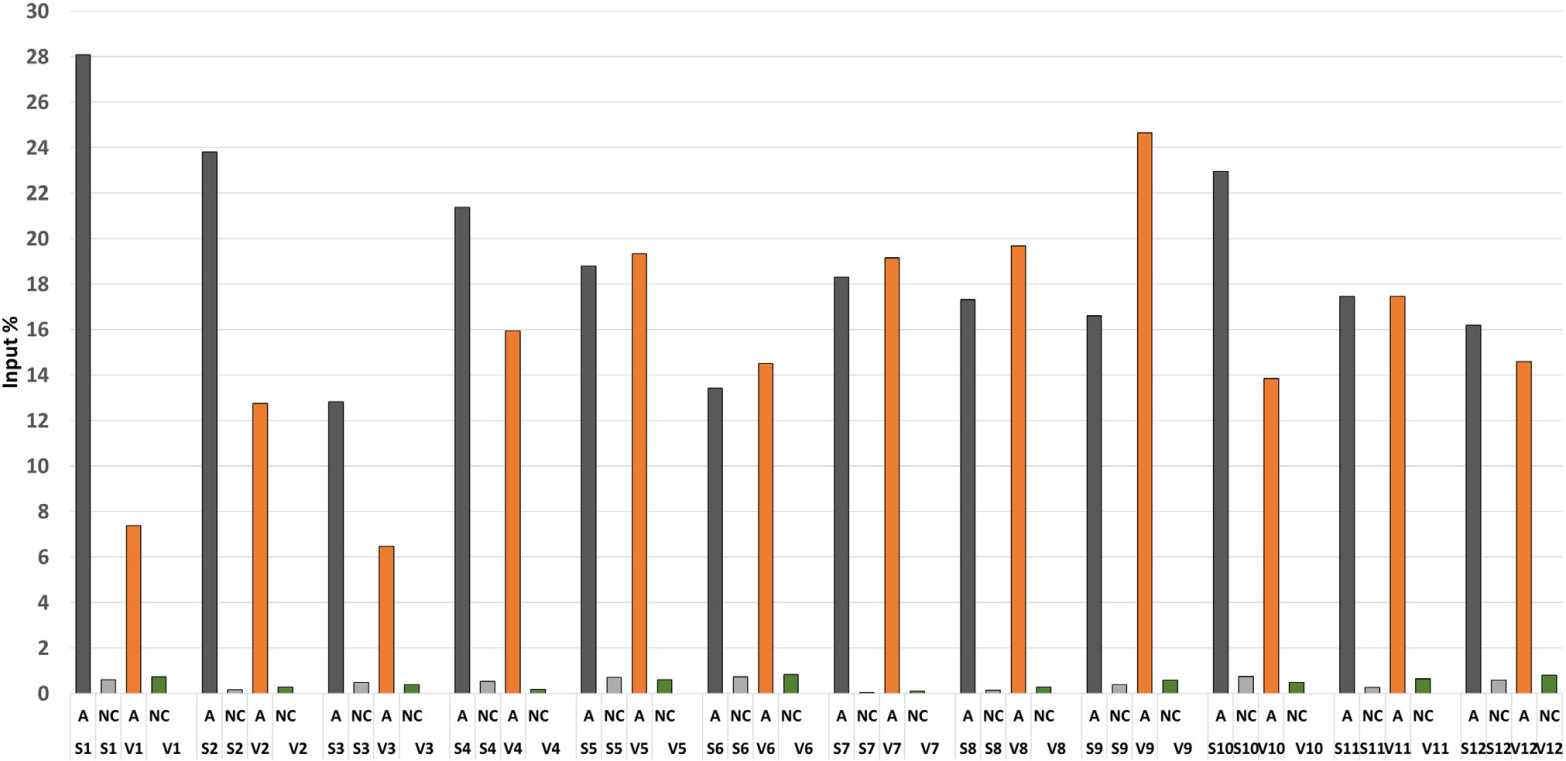
Results of RT-qPCR analysis of two genomic regions across samples of SAT and OVAT (A: actin gene, NC: Human negative control (commercial), S: Sat, V: OVAT)

To assess the success of the ChIP protocol, we recommend employing a target-specific enrichment approach via RT-qPCR. This involves enriching a specific genomic region with H3K36me3, serving as a positive control for an active genomic region (Table 1), and another specific region serving as a negative control (Table 2). For the positive control, we propose a genomic region in the Actin gene and ADIPOQ gene, while a primer set from Active Motif (Human negative control primer set 1) is utilized for the negative control, targeting a gene desert on chromosome 12 (Figure 2). We perform triplicate RT-qPCR runs for each sample and input and then calculate the mean Ct value per sample. To determine chromatin enrichment (% input), we initially apply a logarithmic transformation (log2) to 20-fold (100/5) as it is calculated as 4,321928. Then, we calculate adjusted 100% IP = Ct (Input) - 4,321928. Subsequently, we compute delta Ct as the difference between Ct values of the adjusted 100% IP and the samples. Finally, we calculate the percentage of input (% input) using the formula: 100 ∗ 2ˆ(Delta Ct)."

**Table 1:** qPCR reaction master mix for positive region

Reagent	Amount
2× SYBR Power up master mix	2.5 μL
Primer Fwd/Rev (5 pmol/μL) of actin gene	0.2 μL
Nuclease-free water	0.8 μL
Template (Immunoprecipitated DNA)	1.5 μL

**Table 2:** qPCR reaction master mix negative region

Reagent	Amount
2× SYBR Power up master mix	2.5 μL
Primer (Human negative control)	1 μL
Template (Immunoprecipitated DNA)	1.5 μL

### Sequencing library preparation

**Timing: 120 min**

In this section, we perform library synthesis.

Utilizing ChIP/INPUT materials from samples, we generate a sequencing library for subsequent sequencing, constituting a pool of DNA fragments with specific adapters designed for sequencing. We adhere to the guidelines provided by a commercial kit (Takara Bio USA ThruPLEX DNA-Seq). The library preparation involves three main steps: template preparation, library synthesis, and library amplification. While we do not delve into all details of these steps, which are available in the kit’s guideline, we emphasize on several critical considerations for a successful library preparation:

***Note:*** Thermal cycler conditions, including a heated lid set to 101°C–105°C and ramp rates ranging from 3°C/s to 5°C/s.

***Note:*** The amount of ChIP material in TE buffer as an input buffer (10 mM Tris, 0.1 mM EDTA, pH 8.0). We utilize 0.20 ng of total ChIP/INPUT material for library preparation.

***Note:*** The cycle number in the library amplification step. We employ 15 cycles for this stage.

***Note:*** These conditions are suitable for the H3K36me3, H3K4me3, H3K4me1, H3K27me3, and H3K27ac modifications.

For the sequencing library preparation, we employ the DNA Unique Dual Index Kit (Takara/AH Diagnostics), incorporating index primers carrying the 8-nt long index sequences "IDT for Illumina TruSeq DNA." It’s noteworthy that the kit recommends using input DNA in the range of 50 pg to 50 ng as starting material. We successfully perform library synthesis using 0.20 ng of total ChIP/INPUT material in a 10 μL final volume.

### Library purification by AMPure XP beads

**Timing: 30 min**

Here, we remove small fragments less than 100 bp.

Following library preparation, we employ Agencourt AMPure XP beads (Beckman Coulter, USA), known for selectively binding to PCR amplicons with a size of 100 bp and larger. By following the protocol (000387v001), we successfully generate a sequencing library containing amplicons exceeding 100 bp. To assess the library’s quality, we utilize the 2100 Bioanalyzer system (Agilent Technology, USA) with a high sensitivity DNA kit. The electropherograms obtained for two adipose tissue samples and their INPUTs indicate that the library’s size is optimal for sequencing (Figure 4).

## Expected outcomes

Our specific protocol evolves through modifications of key steps from established ChIP protocols. Given the diverse cellular composition and structure inherent in adipose tissues, our protocol undergoes optimization to ultimately achieve high-quality sequencing data for various histone modifications in both tissue depots concurrently.

### Chromatin concentration after isolation process

The established protocol utilizes a minimal quantity of frozen human adipose tissues. According to our protocol, approximately 100 mg of adipose tissue is anticipated to produce around 2000–2500 ng of chromatin in total. It’s noteworthy that we observe a higher chromatin concentration in OVAT samples (13.43 ± 8.26 ng/μL, *n* = 12) compared to SAT samples (8.81 ± 5.84 ng/μL, *n* = 12). In summary, using 100 mg of either SAT or OVAT should provide sufficient chromatin material for the ChIP process.

### Sonication

We anticipate obtaining high-quality and properly fragmented chromatin from human adipose tissue. As we observe, there is a notable variation between samples originating from different depots, particularly among intra-individually paired samples. Consequently, we foresee the protocol’s capability to generate appropriately fragmented chromatin (mainly ranging between 200-600 bp) for all individuals. Through extensive testing under various conditions, we determine that 8 cycles, repeated 4 times (32 cycles), result in optimal chromatin fragmentation (Figure 1). Following each set of 8 cycles, the samples are mixed and kept on ice for 2 min before proceeding with the subsequent cycle. It’s worth noting that OVAT tissue samples may exhibit variations in response to sonication conditions, potentially necessitating additional sonication.

### Chromatin immunoprecipitation

**Figure 3:**
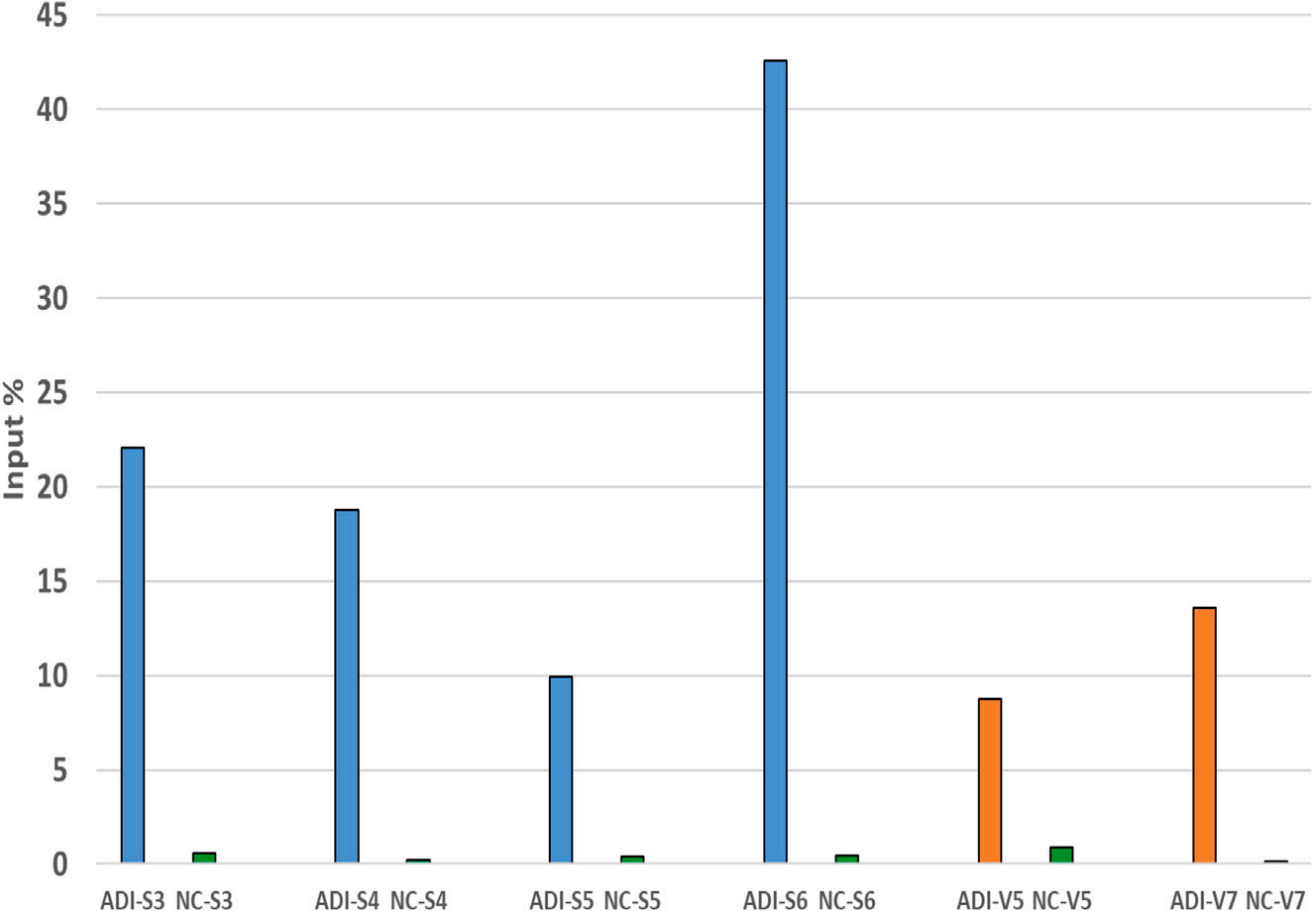
Results of RT-qPCR analysis of ADIPOQ gene across samples of SAT and OVAT (ADI: ADIPOQ gene, NC: Human negative control (commercial), S: Sat, V: OVAT)

We systematically test various conditions, including different bead types, antibodies, and incubation times for beads and antibodies, as well as chromatin, to eliminate non-specific binding while achieving high-quality enrichment of the positive genomic region. The protocol is validated for 400–500 ng of chromatin. While theoretically, 25 mg of tissue might suffice for the ChIP process, we recommend starting with 100 mg of frozen adipose tissue. Despite the protocol requiring a relatively low concentration of chromatin when compared with previous protocol,^2^^,^^3^ we anticipate achieving higher enrichment of the positive control (Figures 2 and 3). In summary, the ChIP/Input chromatin obtained for each sample is sufficient for q-PCR analysis and subsequent library preparation.

### Anticipated

#### Expected RT-qPCR results

Prior to sequencing, it’s imperative to validate the protocol’s efficacy. Hence, RT-qPCR serves as a critical checkpoint to ascertain its success. To evaluate the effectiveness of each procedural step, we select two genomic regions: one rich in H3K36me3 and a commercial negative control. We assess enrichment via RT-qPCR to gauge the coherence of the process, calculating IP% based on the obtained data. Ideally, we anticipate negligible enrichment (around 1% or less) for the negative control and substantial enrichment for H3K36me3. Elevated enrichment in the negative control would suggest non-specific binding. Conversely, in case of H3K36me3, obtaining∼ 10% IP or more in the positive region indicates a successful enrichment. In our experiment, employing the refined protocol on human SAT and OVAT samples from 12 subjects, we observe higher enrichment in the negative control, while achieving the desired enrichment in the positive region, paving the way for sequencing."

#### Quality of library

**Figure 4:**
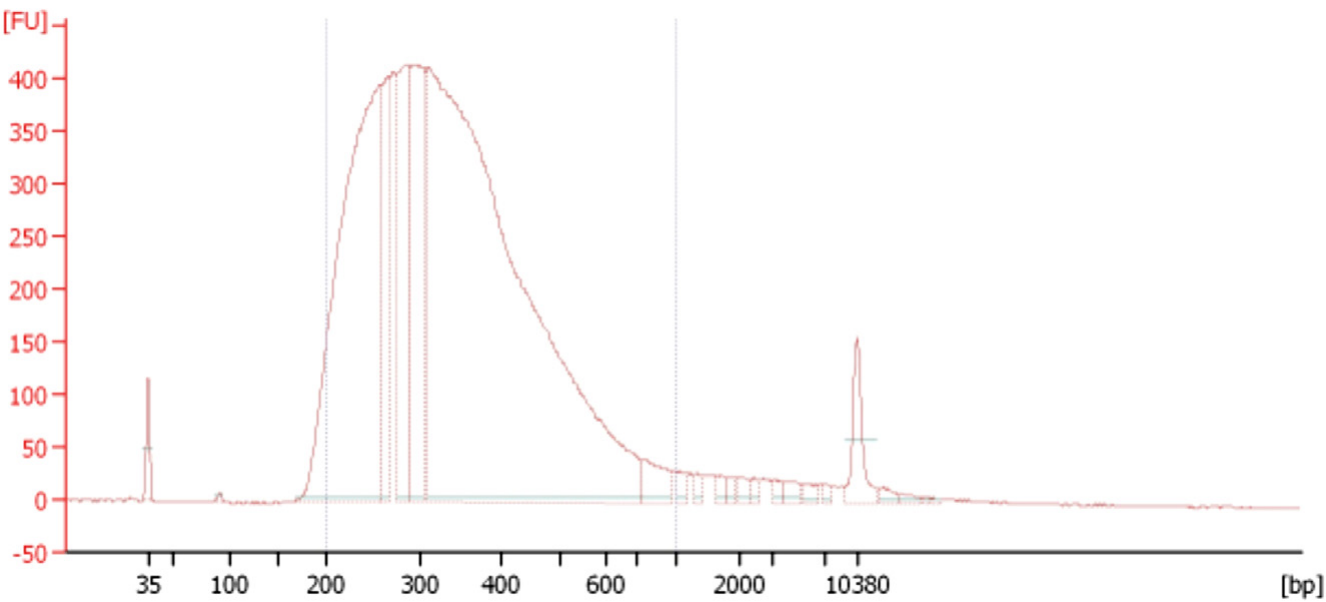
An example of library generated from SAT tissues using 0.20 ng of immunoprecipitated chromatin

We expect to obtain high quality of library. We obtain libraries with high quality; proper chromatin fragment sizes and sufficient quantity for sequencing after preparing 12 samples of SAT and OVAT (Figure 4).

## Limitations

This protocol is designed with the following objectives: **1)** to achieve a higher chromatin yield, **2)** to conduct successful immunoprecipitation with minimal nonspecific background, **3)** to enable successful high quality library synthesis, and **4)** to produce high-quality sequencing data for H3K36me3. We also apply the protocol’s applicability to H3K4me3, H3K4me1, H3K27me3, and H3K27ac modifications by testing various antibody incubation times with beads and the antibody-beads complex with chromatin. We obtain successful results. While each step of the protocol undergoes thorough testing, it’s important to note that when targeting different modifications or proteins, critical parameters include antibody type, incubation with beads, and chromatin interaction. These parameters are modification-specific and require precise adjustments.

Notably, the protocol is not validated for cells from cell culture (e.g., pre-adipocytes or mature adipocytes) or non-adipose tissues. Therefore, optimization for specific cell types or tissues may be necessary.

## Troubleshooting

The primary goal of this protocol is to ensure successful immunoprecipitation, library synthesis, and the generation of high-quality data. Successful tests are conducted for H3K36me3 histone modification. More importantly, the protocol shows successfully application for additional four distinct histone modifications in human SAT and OVAT samples.

### Problem 1

Variation in Chromatin Amount.

It expects due to variations in chromatin quantity among individual samples in a population study, especially with some samples exhibiting lower concentrations.

### Potential solution

The lowest concentration obtained in our study (*n* = 12) is sufficient for ChIP and INPUT. For potential experiment repeats, we suggest the use of over 100 mg of tissue for samples with low chromatin amounts.

### Problem 2

Sonication Efficiency in OVAT Samples.

Varying efficiency in sonication, leading to varied fragment sizes in OVAT samples.

### Potential solutions

• After testing different conditions, an optimal solution is applying 5 × 8 = 40 cycles of sonication instead of 4 × 8 = 32 cycles to achieve the proper size.
• The use of Covaris ultrasonicator can be beneficial, with testing required for each Covaris conditions to eliminate variations.

### Problem 3

Type of Antibody and Concentration.

Issues related to the type of antibody and its concentration, especially when the protocol is employed for different targets, such as diverse histone modifications or transcription factors.

### Potential solution

Testing antibodies from various companies and initially considering the concentrations suggested by the manufacturers.

### Problem 4

Incubation Time of Antibody with Beads and Chromatin.

Critical challenges in determining the appropriate incubation time with beads and the incubation time of the antibody-beads complex with chromatin.

### Potential solution

Finding the optimum incubation time, such as observing that 1 h is suitable for some histone modifications. It is critical that extended incubation times, like overnight, can increase unspecific binding of negative control.

## Resource availability

### Lead contact

Further information and requests for resources and reagents should be directed to and will be fulfilled by the lead contact, Yvonne Böttcher (yvonne.bottcher@medisin.uio.no).

### Technical contact

Further information and requests for technical issues should be directed to and will be fulfilled by the technical contact, Akin Cayir (akin.cayir@medisin.uio.no).

### Materials availability

This study did not generate any materials.

### Data and code availability

This study did not generate new code. Original data are shown in the figures. ChIP-seq data are not part of the ChIP protocol and therefore not shown.
